# Building social capital to promote adolescent wellbeing: a qualitative study with teens in a Latino agricultural community

**DOI:** 10.1186/s12889-017-4110-5

**Published:** 2017-02-08

**Authors:** Marissa Raymond-Flesch, Colette Auerswald, Linda McGlone, Megan Comfort, Alexandra Minnis

**Affiliations:** 1 Department of Pediatrics, Division of Adolescent and Young Adult Medicine, University of California San Francisco, 3333 California Street, Suite 245, San Francisco, CA 94118 USA; 2 School of Public Health, Division of Community Health Sciences, University of California Berkeley, 570 University Hall, Berkeley, CA 94702 USA; 3 Monterey County Health Department, 1270 Natividad Road, Salinas, CA 93906 USA; 4 Division of Behavioral Health and Criminal Justice, Research Triangle Institute International, 351 California Street, Suite 500, San Francisco, CA 94104 USA; 5 Women’s Global Health Imperative, Research Triangle Institute International, 351 California Street, Suite 500, San Francisco, CA 94104 USA; 6 University of California Berkeley School of Public Health, 351 California Street, Suite 500, San Francisco, CA 94104 USA

**Keywords:** Adolescent, Latino, Social capital, Protective factors, Teen pregnancy, Youth violence

## Abstract

**Background:**

Latino youth, particularly in rural settings, experience significant disparities in rates of teen pregnancy and violence. Few data are available regarding social and structural influences on Latino youth’s developmental trajectories, specifically on factors that promote wellbeing and protect them from engagement in high-risk sexual and violence-related behaviors.

**Methods:**

Forty-two youth aged 13 to 19 years old were recruited from middle schools and youth leadership programs to participate in one of eight community-based focus groups in Salinas, a predominantly Latino, urban center in California’s rural central coast. Focus groups covered youths’ experiences with the risk and protective factors associated with exposure to violence and romantic relationships. Four researchers completed coding with a Grounded Theory approach, informed by the theoretical frameworks of the social ecological model and social capital. The study’s design and participant recruitment were informed by a community advisory board of local youth-serving organizations and health care providers.

**Results:**

Participants described family lives rich in bonding social capital, with strong ties to parents and near-peer family members. They reported that while parents had a strong desire to promote healthful behaviors and social mobility, they often lacked the bridging or linking social capital required to help youth navigate structural systems, such as college applications and access to confidential health care. Youth also reported that some families link their children to negative social capital, such as exposure to gang affiliation.

**Conclusion:**

Adolescents in this agricultural community identified robust sources of bonding social capital within their families. However, they identified limitations in their families’ capacities to link them to structural resources in education, employment, and health care that could support healthful behaviors and upward social mobility.

## Background

Latinos will comprise one-third of United States adolescents aged 10 to 19 by the year 2040 [1, 2]. Compared to non-Latino youth, Latino adolescents experience numerous health disparities including a higher prevalence of teen pregnancy and youth violence [2–4]. While poverty and immigration likely contribute to these disparities [5], an understanding of protective factors conferred by immigrant families and communities is also essential to promoting youth wellbeing [6].

There is growing evidence that youth’s resilience and health outcomes arise not only from individual traits, but also from the influences of families and communities [7]. Social capital, defined as a “wide variety of quite specific benefits that flow from the trust, reciprocity, information and cooperation associated with social networks,” may explain, in part, how family and community networks shape the health and health behaviors of adolescents [8]. Subtypes of social capital, including bonding, bridging, and linking social capital, may benefit youth via different mechanisms [9–11]. *Bonding social capital* comprises trusting relationships between members of a social group who share a common social identity [11]. In the case of adolescents, bonding social capital might include peers, partners, siblings, or other family members. *Bridging social capital* refers to respectful relationships and mutuality between those who are not alike in some aspect of their socio-demographics (e.g., age, ethnic group, socioeconomic status) [11]. In the case of adolescents, bridging social capital could include adults in their community such as teachers, counselors, religious leaders, or health care providers [12]. Finally, *linking social capital* consists of networks of trusting and respectful relationships between people who are acting across explicit power or authority gradients [11]. In the case of adolescents, linking social capital might include relationships that promote youth connection with institutions outside of their immediate community such as decision-makers in higher education, employment or government.

Researchers have examined the influence of multiple sources of social capital on adolescent health behaviors [13, 14]. This ecological conceptualization reflects the contributions of multiple dimensions of an individual’s environment to behavior [13–16]. A recent systematic review of research on social capital and adolescent health behaviors found that family social capital, reflected in the quality of parent-child engagement and presence of two parents in the household, is associated with lower rates of substance use, delayed sexual activity, increased contraception use, and higher educational attainment [13, 17–22]. Another systematic review found that family and community social capital affected adolescents’ externalizing behaviors, including violence and aggression [14]. Risk of externalizing behavior increased for adolescents who reported low family social capital (parental support) and lower social cohesion in school [23]. In aggregate these studies provide a compelling case that social capital warrants additional investigation and may be a target for future interventions. As social capital may operate differently across communities, it is particularly important to understand its specific forms and effects on Latino youth to guide culturally-informed interventions. Furthermore, few studies have examined how social capital influences developmental trajectories for youth residing in non-urban settings.

The aim of the *A Crecer* study is to use a community-engaged, approach to investigate the structural and interpersonal factors that influence developmental trajectories related to school engagement, hope for the future, resiliency, onset of youth violence and sexual health risks for Latino youth living in an agricultural community as they transition from early to middle adolescence. Prior research on the developmental trajectories of urban Latino youth suggests that Latinos may differ from other populations of adolescents. One longitudinal study on the risk and protective factors related to gang involvement, for example, suggested that Latino youth were less susceptible to influence from peers engaging in deviant behaviors, but may be more susceptible to substance use than their white counterparts [24]. Other research with urban youth has shown that family engagement has a larger protective effect on substance use and recidivism among Latino juveniles incarcerated for gang-related activities than for their white peers [25, 26]. Issues related to acculturation and immigration can also uniquely affect the developmental trajectories of Latino youth, with some data suggesting that recent urban immigrants may be at higher risk for unintended pregnancy [27]. Immigration in agricultural communities affects family structures and relationships as well as neighborhood environments that support youth. This paper presents the results of the baseline qualitative phase of *A Crecer*. We examined sources of social capital through the voices of Latino youth in this agricultural community, as well as youth’s narratives regarding how social capital constrains and/or promotes positive development and health outcomes, potentially leading to different adolescent trajectories.

## Methods

### Setting

Salinas, California is a small urban center in the heart of California’s agricultural central coast. The county of Monterey, where Salinas is located, has the seventh highest rate of teen births of the 58 counties in California. Ninety-three percent of these births are to Latinas [28, 29]. The county also has the state’s highest rates of teen hospitalizations for assault and teen homicide, driven in large part by high rates of regional gang violence [2, 4]. These poor health outcomes for adolescents are further complicated by the socioeconomic disparities associated with low-wage agricultural employment in the region [30]. The middle schools from which we recruited youth reflect the predominantly Latino population of Salinas, with Latino students constituting 84 to 98% of the student body across schools [31]. Socioeconomic disadvantage, as measured by eligibility for free and reduced lunch, is high, ranging from 72 to 93% of the student population across schools [31].

### Study design

We designed *A Crecer* using a community-based research approach with community partners engaged in all aspects of research design and implementation. *A Crecer* was initiated when the Monterey County Health Department approached the study’s principal investigator to discuss the community’s interest in investigating the roots of these health outcomes and in characterizing strategies that the community can use to support school engagement, resiliency, and hope for the future among Salinas youth. The study team developed a community advisory board of seventeen local youth-serving organizations as well as a youth advisory board. *A Crecer* is also informed by partnerships with school principals and parents in the Salinas Unified School District. While adults in the Salinas community contributed to *A Crecer*’s development and implementation through parent meetings, key informant interviews, and the study’s community advisory board, we conducted focus groups to formally engage additional youth in the formative phase of our study.

We conducted six gender-stratified focus groups with eighth-grade students recruited from three Salinas middle schools. We completed two additional focus groups with middle school and high school students engaged in two community-based youth leadership programs. While most of our focus groups were stratified by gender, one of the focus groups that we completed with a community-based youth leadership program was a mixed gender group. We conducted this group to recruit a larger number of newly immigrated students as participants, which contributed to the diversity of our sample. None of the youth participated in more than one focus group. Bilingual study staff conducted recruitment in schools and at parent meetings, employing bilingual study materials.

Eligibility criteria included living in Salinas, ability to speak English, signed participant assent, and signed parental permission. A bilingual field coordinator spoke in-person or by telephone with participants’ parents to address questions and discuss the project. In accordance with community advisory board input and based on our assessment of schools, the number of monolingual Spanish-speaking teens at targeted schools was found to be low. Given the low numbers of Spanish-speaking only students and the relatively high number of later generation immigrants who preferred to speak in English, focus groups were conducted in English, though participants were invited to express themselves in Spanish if they preferred.

### Data collection

Focus groups were 60 to 90 min long and took place at local community settings. We targeted recruitment of four to ten participants per group based on our pilot group and an average of five youth participated in each group. While bilingual moderators were present and focus group participants occasionally used Spanish, groups were conducted primarily in English. We pilot tested the focus group guide with a group of 8th graders. Following pilot testing we shortened the moderators guide to improve pacing; refined prompts to promote discussion of ideal social experiences and actual experiences, particularly related to gender differences; and removed a closing activity due to low participant energy at the end of the group.

**Fig. 1:**
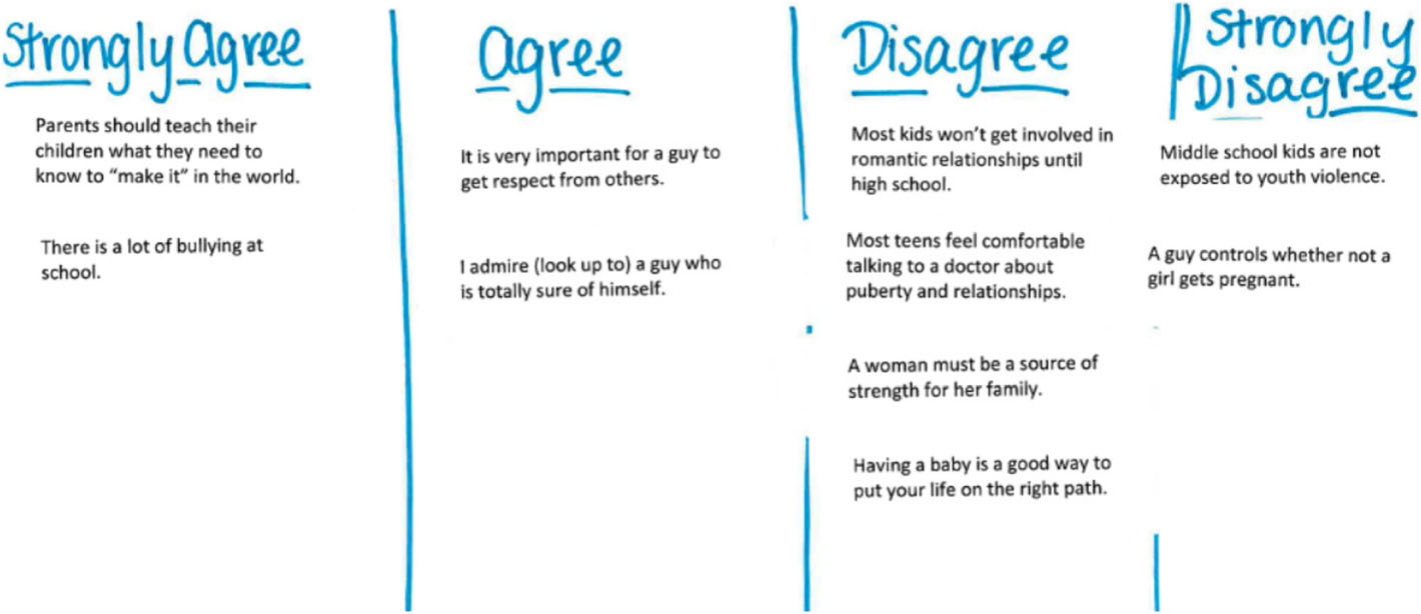
In the first focus group activity, participants used stickers to rank their relative agreement/disagreement with statements about family, gender, and relationships. Participants then discussed their opinions with the group

**Fig. 2:**
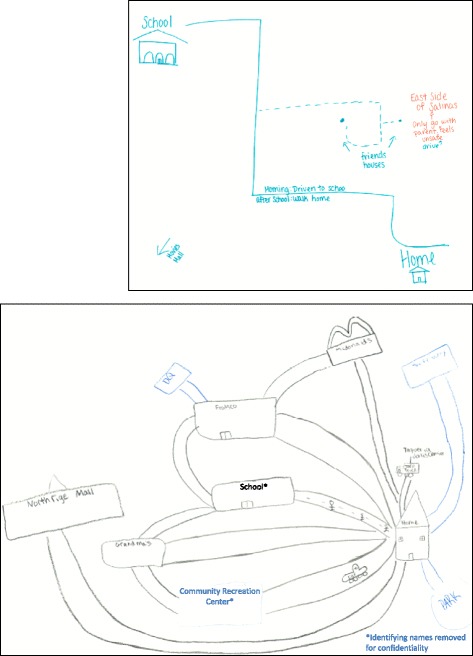
In the second focus activity, participants identified structural and neighborhood factors that affected their mobility, access to community resources and sense of safety. In this activity participants drew a map of their community including school, home, recreational settings, transportation methods, and places where they felt safe/unsafe and discussed their maps with the group. *The names of identifying community locations were changed to protect participant privacy

The lead moderator was a Latina adolescent medicine physician experienced in focus group moderation. Local study team members acted as assistant moderators. The focus group activities and moderator’s guide were developed using the social ecological model, the organizing framework for the overall study [15, 16]. Activities were designed to encourage youth to speak about the interpersonal and community-level norms and influences that they perceived as important in the lives of Salinas youth. In one activity, participants were given a sheet of 10 labels, each with a pre-printed statement reflecting a behavioral norm tied to one of the study outcomes or social/structural factor hypothesized to influence the outcomes (e.g., “Most kids won’t get involved in romantic relationships until high school.”). Youth were asked to indicate their level of agreement with the statement by placing the label under a column heading (i.e., 4-point Likert scale that ranged from “Strongly Agree” to “Strongly Disagree”) that aligned with their individual view (see Fig. 1). After youth had labeled each statement, the moderator facilitated a discussion about each norm, drawing out diverse perspectives and rationale for what motivated the participant’s level of agreement. The second activity was designed to focus on neighborhood environment, first and foremost, and involved drawing individual neighborhood maps that included places youth spend time, mobility, neighborhood assets (e.g., parks, recreation centers), and safety concerns (see Fig. 2). Through both activities, the discussions addressed sources of support for youth, violence exposure, onset of romantic relationships, community engagement, and the impact of gender and ethnicity on youth’s experiences. For the specific prompts that we used to facilitate group discussion and guide participant activities see our moderators guide. Participants were compensated with a snack and $10 in cash. The Institutional Review Board at RTI International approved all study procedures.

### Analysis

Four researchers participated in the qualitative analysis, including identification of themes, development of a codebook, and memo writing. All transcripts from the focus groups indicated the gender composition of the group and the recruitment location, which permitted us to take those contexts into account in the analysis (e.g., noting whether statements expressing certain views were typically made by males or females). The same codebook was used for all transcripts were analyzed via the same process. Each transcript was coded by two team members with a third team member reviewing codes for consistency. Team members completed memos in two rounds. Initially each team member drafted informal memos about reoccurring themes in that arose in their coding. In a second round of memos team members created formal documents that were drafted and revised by multiple team members on themes and codes of interest including social capital, safety, gender roles, and family connections. The team used these memos to refine our coding, deepen our analysis, and inform the next phase of the *A Crecer* study (e.g., refine research questions and revise quantitative instruments for future phases of the study).

Consistent with grounded theory analysis [32, 33], codes were primarily drawn from the data. While the concept of social capital was not integral to our original theoretical framework, it emerged through this iterative coding process as a core category. For the purpose of this analysis (and consistent with existing social capital literature), bridging and linking social capital were collapsed as the boundaries between them blurred in youth’s narratives. Preliminary findings were presented to the study’s community advisory board to contextualize and validate findings.

## Results

Forty-two Salinas youth (85% Latino; 55% female) participated in one of the eight focus groups. Two-thirds were 13 or 14 years old and the remainder were 15 to 19 years old. Twenty-seven youth participated in eighth-grade only groups and 15 were drawn from existing community-based youth leadership programs. Nearly all reported speaking both English and Spanish at home. We present participants’ description of their families’ contribution to their social capital (bonding capital), discuss their extra-familial sources of social capital (bridging/linking social capital), note limits to youth’s social capital, and illustrate how social capital affects youth as they navigate the social ecological contexts influencing their health.

### Families Constitute a Primary Source of Bonding Social Capital

Youth reported that they spend most of their non-school time close to home with parents and relatives, turning to family members for advice preferentially over friends. They emphasized the important and different roles of parents and same gender near-peer relatives such as siblings and cousins. Many youth described how critical their parental relationships are to their well-being, motivation for academic achievement, and life guidance. A male eighth grader said, “If parents don’t teach [their children] anything, they can go to the wrong path, or they might just like get into gangs.” Another eighth grade male participant reported,

*I admire my dad, who spends his time working, but at the same time has the time to spend with his family, and he’s given me a lot of advice… [H]e works hard to buy things that I want and just keeps me happy.*

Other participants, such as this male eighth grader, described the pivotal guiding role a single-parent played,

*My dad was never there …My older brother …was already getting into gangs. My sister was barely getting out of elementary, so it was difficult. But we got through that all because of my mom. [My brother is] now at [one] university. My older sister is at [another], and then I’m next.*

Bonding with parents was so strong that some participants reported longing for more engagement with their parents, who were often preoccupied with the challenges of supporting their families. One female high school student said,

*I feel like some parents really are into the work industry, “Oh, I have to go to work tomorrow.” Or like, “I have to make food.”…And they don’t really pay attention to like, “Oh, I wonder how her day was,” or, “I wonder what’s going on with her and her boyfriend.”*

Youth suggested that even when parents had made life choices that they did not want their children to repeat, they could still draw on these experiences to provide guidance to youth. One male participant explained, “Parents teach [their children] what the parents went through, like if they got pregnant at an early age, how like they messed up, and like challenges they faced.” Similarly, even if parents lacked their children’s educational opportunities they can still encourage their children’s school engagement. One male youth leadership group participant explained,

*I think parents should teach their kids to…find the path in their life…For example, if I’m Mexican…maybe [I would] think, “Oh, maybe I need to go to the fields to work because my parents tell me,” or something like that. But if the parents say, “Oh, no, you know, mijo, you need to go to college to have a better life,” that may be other motivation for the kids*.

In addition to their parents, youth’s siblings and cousins were cited by participants as a critical source of support. One eighth grade male recounted,

*I talk about my brother a lot but—he’s the only one that taught me things…He would tell me, you just want to be good ‘cause colleges would be lookin’ at you. So you don’t really want a girlfriend freshman and sophomore year…. You want to get to college, and then later think about a girlfriend.*

Participants reported that they prefer to talk about romantic relationships and reproductive health with near-peer family members, followed by friends or medical professionals. One eighth-grade girl explained that she could discuss puberty with, “people around your age. Like, my cousin and my sister, because they understand me, I understand them.” Thus near-peer family members acted as role models for educational pursuits and romantic relationships.

### Bridging and Linking Social Capital: Accessing Resources Outside of the Family

Although participants described that parents played a critical role in supporting youth through bonding social capital, they also recognized that parents were not always able to guide children who were growing up under circumstances vastly different from their own upbringing. Parents’ limited access to social opportunities and limited bridging networks can hinder them from directly linking youth to educational and health resources. However, as one eighth grade male participant explained, bridges to other social resources, such as teachers, can help parents overcome this challenge:

*The parents may need to be taught first about what they need to do, what’s right for the kids. So having a teacher to help them out and teach their kids, all the while teaching to the parents, could help them.*

A female high school student in one of the youth leadership groups explained that many adults were supporting Salinas youth to make the most of their lives, “Like any other community, we’re not perfect—so the community’s kind of involved in high school students and trying to better us.”

Some participants noted that teachers can help students who are engaged in unsafe behaviors, for example, encouraging students to leave gangs and pursue higher education. One eighth grade male participant explained that his brother had struggled in middle school but began to thrive in high school:

*The only reason [my brother] started being smarter is because of his peers, his teachers. He had friends that were gang members, [who] even told him that he shouldn’t be in this, he’s smarter than that … and he’s doin’ good now. He’s commuting to [college].*

Other participants pointed out a key role that schools play in linking youth to health education programs to teach them about pregnancy prevention and reproductive health issues when families may not be comfortable or equipped to address these topics. One high school female explained,

*When I was in middle school, I think three girls were pregnant at that time, and then I think I was the first class that had a health class in middle school, ‘cause I guess our school saw the problem, that kids were beginning to deal with these relationships,…and then your parents don’t really—they’re not open about that process.*

### Limits to Social Capital

Although other adults may be able to coach parents or fill in where parents’ experiences are limited, the differences between an adolescent’s experiences and their parents’ can nevertheless make it difficult for youth to navigate the range of opportunities for the future. Parents may not be equipped to link their children to needed social capital resources, or may inadvertently miss opportunities to link youth to social capital. One eighth grade girl explained, “It’s like [parents] tell them that they have to go to school so they can get a better life and stuff…but they can’t teach ‘em how to get to college if they don’t do it for themselves.” Another female participant from a high school youth leadership group explained that she wanted more support from her parents in her educational pursuits, but that their expectations were often unrealistic.

*Personally, I’m not able to discuss things that happen during school, or even if it’s just like a scholarship or something, ‘cause I understand that they didn’t have as many opportunities as we have, so they want us to go after a lot of ‘em. But sometimes it can be really stressful…they sometimes expect maybe a little too much.*

Similarly, differences in romantic and cultural norms between immigrant parents and their children can have a negative impact on the health of youth by limiting access to bridging capital, in this case their physician. One female participant in a youth leadership group explained that her parents’ expectations regarding her medical care limited her access to important reproductive health information.

*“I feel like having Mexican parents, they usually go in the room with you when they [the doctors] check you so you can’t really talk. That has happened to me a lot before.”*

Another female participant from a high school youth leadership group described how parental attitudes about romantic relationships and sex made it hard for youth to access parents (bonding capital) or resources (linking capital) to prevent unwanted pregnancy:

*I believe, as females in the Mexican culture, it’s a lot harder to speak to your parents about relationships…They’re afraid of us getting pregnant or leaving and they tend to push us away and not tell us experiences they’ve had… I believe that many females, young females who do end up pregnant as teenagers, I think it’s because of that—[they are] afraid to speak about relationships and sex.*

Shifts in technology have also widened the gap between parents and youth, limiting the capacity for parents to offer bonding social capital. One female eighth grader explained what happened when her half-sister got a smartphone.

*She just did like a 180. Totally different person—the crowd she hangs out with, really bad… [Her mom’s] not in touch with a lot of it, so she doesn’t know what’s on there. I think a lot of parents kinda are blind-sided to the fact that kids nowadays are more tech-savvy*.

### Potential Links to Negative Social Capital

Participants reported that in some families adolescents were encouraged to employ aggressive strategies for problem solving, or to access community networks that engage in violence, such as gangs. One female high school participant explained,

*I don’t know if it’s just my family or if it’s like a culture thing,…[but] if you have a problem, go fix it by violence, go fight them. And if you don’t, or if someone tries to fight you, and you don’t want to fight them, it’s kinda sayin’, like you’re a coward.*

Others noted that gang affiliation can be passed from one family member or one generation to another. In these cases linking social capital serves to foster gang membership, as one eighth grade male participant described: “It’s like a chain, that if someone has an older sibling that’s involved in a bad choice, for example, gangs, that the younger sibling will continue with that because he gets introduced to it by the older sibling.”

## Discussion

Middle school and high school students in the predominantly Latino, agricultural community of Salinas, California describe parental relationships rich in bonding social capital that support interpersonal relationships, promote inner strength, and encourage them to avoid high-risk behaviors like gang involvement. Youth also shared the key role of near-peer relatives who further support their bonding social capital. However, youth describe their families’ limited capacity to provide bridging/linking capital to community resources such as higher education, confidential health care, and upwardly mobile jobs. They reported a need for other adults in the community, such as educators and health care providers, to link youth to these resources and to support parents in furthering those connections.

Our results are supported by prior findings with general adolescent populations which identified parents as an important source of social capital protecting youth from high-risk sexual activity, substance use, and externalizing behaviors [13, 17–23, 34]. Our work extends these findings to a rural immigrant Latino population. While prior studies have described the importance of non-parental adults (e.g., grandparents) in reducing health risk behaviors [35, 36], our results add to these findings by also emphasizing the key role of near-peer relatives in helping adolescents navigate puberty, reproductive health, educational challenges, and gang affiliation.

Limits of parental social capital among immigrant and minority families have also been reported within the educational literature, with families struggling to promote educational attainment due to limited social ties and language discordance [37]. However, as suggested by the participants in this study, research has demonstrated that educational linkages can be supplemented by other adult role models such as mentors and guidance counselors [36]. Schools can also train parents, bolstering their capacity to link their children to higher education resources, regardless of parent educational attainment [38]. In some cases, as illustrated in this study, youth note that without this guidance parents may only have the capacity to link them to their own limited resources, which in some cases may include low-income employment or gang affiliation. These sources of negative social capital add to a growing literature about the ways that social capital can limit social or economic mobility or cause harm [39].

Our findings have several limitations. Although data saturation was achieved, the sample in this study is small, limiting its generalizability. Focus groups were conducted predominantly in English which may have excluded some perspectives, particularly those of the newest immigrant community members. While focus groups can promote discussion of group norms, some participants may be hesitant to discuss sensitive topics or disclose controversial or differing viewpoints with peers. We conducted recruitment in schools and organized extracurricular settings. Although every effort was made to achieve a diverse sample, youth who were most at-risk (those who are out of school or for whom we were unable to obtain parental permission) are not represented here.

## Conclusion

This study illustrates the ways in which multiple types of social capital impact on youth health behaviors and developmental trajectories. In this predominantly Latino population it is important to acknowledge the strong foundation of bonding social capital between youth and their parents and near-peer family members. Indeed, our findings suggest that further research is merited regarding the health effects of near-peer family members, particularly given their potential to provide support to youth in families that are faced with economic strain, or separated as a result of parental migration, employment, incarceration or divorce. Given that adolescents in single-parent households are at increased risk of substance use, high-risk sexual activity, and violent externalizing behaviors [13, 40], near-peer family members may be appropriate targets for adolescent health promotion programs to extend bonding social capital for early adolescents in at-risk communities.

In addition, this study highlights how parents’ resiliency can foster the resiliency of their adolescent children. In many ways, the parents’ immigration is itself a tremendous act in the pursuit of bridging/linking social capital, and the strongest contribution that they can make in this regard to their children. While these families often lack further forms of bridging and linking social capital within the United States, some initiatives, such as the California Endowment’s Building Health Communities [41], work beyond families to strengthen extended community networks. Parents’ capacity for bonding social capital is foundational to the sense of safety that, in this study, allowed youth to aspire to educational attainment, healthful choices, and social mobility. Family-based interventions that build positive parenting skills, address harsh parenting styles, and work to strengthen relationships between parents and their youth can promote bonding capital. Several family-based interventions for parents and early adolescents have been found to improve adolescent mental health, decrease substance use, delay onset of sexual activity, and increase school engagement [42–44]. Our work suggests the need to support programs that capitalize on the innate bonding capital in these families while building the parental capacity for bridging and linking social capital to higher education, employment, and health care opportunities that will allow youth upward mobility and wellbeing and they transition to adulthood.
